# Effect of *MAOA* DNA Methylation on Human in Vivo Protein Expression Measured by [^11^C]harmine Positron Emission Tomography

**DOI:** 10.1093/ijnp/pyac085

**Published:** 2022-12-27

**Authors:** Patricia A Handschuh, Matej Murgaš, Chrysoula Vraka, Lukas Nics, Annette M Hartmann, Edda Winkler-Pjrek, Pia Baldinger-Melich, Wolfgang Wadsak, Dietmar Winkler, Marcus Hacker, Dan Rujescu, Katharina Domschke, Rupert Lanzenberger, Marie Spies

**Affiliations:** Department of Psychiatry and Psychotherapy, Medical University of Vienna, Austria; Department of Psychiatry and Psychotherapy, Medical University of Vienna, Austria; Department of Biomedical Imaging and Image-guided Therapy, Division of Nuclear Medicine, Medical University of Vienna, Austria; Department of Biomedical Imaging and Image-guided Therapy, Division of Nuclear Medicine, Medical University of Vienna, Austria; Department of Psychiatry and Psychotherapy, Medical University of Vienna, Austria; Department of Psychiatry and Psychotherapy, Medical University of Vienna, Austria; Department of Psychiatry and Psychotherapy, Medical University of Vienna, Austria; Department of Biomedical Imaging and Image-guided Therapy, Division of Nuclear Medicine, Medical University of Vienna, Austria; Center for Biomarker Research in Medicine (CBmed), Graz, Austria; Department of Psychiatry and Psychotherapy, Medical University of Vienna, Austria; Department of Biomedical Imaging and Image-guided Therapy, Division of Nuclear Medicine, Medical University of Vienna, Austria; Department of Psychiatry and Psychotherapy, Medical University of Vienna, Austria; Department of Psychiatry and Psychotherapy, Medical Center – University of Freiburg, Faculty of Medicine, University of Freiburg, Freiburg, Germany; Centre for Basics in Neuromodulation, Faculty of Medicine, University of Freiburg, Germany; Department of Psychiatry and Psychotherapy, Medical University of Vienna, Austria; Department of Psychiatry and Psychotherapy, Medical University of Vienna, Austria

**Keywords:** *MAOA*, DNA methylation, monoamine oxidase A, positron emission tomography, seasonal affective disorder

## Abstract

**Background:**

Epigenetic modifications like DNA methylation are understood as an intermediary between environmental factors and neurobiology. Cerebral monoamine oxidase A (MAO-A) levels are altered in depression, as are DNA methylation levels within the *MAOA* gene, particularly in the promoter/exon I/intron I region. An effect of *MAOA* methylation on peripheral protein expression was shown, but the extent to which methylation affects brain MAO-A levels is not fully understood.

**Methods:**

Here, the influence of *MAOA* promoter/exon I/intron I region DNA methylation on global MAO-A distribution volume (V_T_), an index of MAO-A density, was assessed via [^11^C]harmine positron emission tomography in 22 patients (14 females) suffering from seasonal affective disorder and 30 healthy controls (17 females).

**Results:**

No significant influence of *MAOA* DNA methylation on global MAO-A V_T_ was found, despite correction for health status, sex, season, and *MAOA* variable number of tandem repeat genotype. However, season affected average methylation in women, with higher levels in spring and summer (*P*_uncorr_ = .03). We thus did not find evidence for an effect of *MAOA* DNA methylation on brain MAO-A V_T_.

**Conclusions:**

In contrast to a previous study demonstrating an effect of methylation of a *MAOA* promoter region located further 5’ on brain MAO-A, *MAOA* methylation of the region assessed here appears to affect brain protein levels to a limited extent at most. The observed effect of season on methylation levels is in accordance with extensive evidence for seasonal effects within the serotonergic system.

**Clinicaltrials.gov Identifier:**

NCT02582398 (https://clinicaltrials.gov/ct2/show/NCT02582398).

**Significance Statement**

Changes to methylation of the promoter/exon I/intron I region of the *MAOA* gene were shown in depression, as were changes to brain levels of the corresponding monoamine oxidase A (MAO-A) protein, which is essential for serotonin degradation. However, though *MAOA* methylation was shown to affect expression in vitro, the extent to which methylation within this gene region affects brain levels in humans in vivo, and whether effects differ between healthy and depressed individuals, is unclear. Here, we did not detect a significant influence of *MAOA* promoter/exon I/intron I DNA methylation on cerebral MAO-A levels assessed with [^11^C]harmine positron emission tomography, suggesting that methylation effects are minor in the context of in vivo brain MAO-A variability. However, methylation levels varied across the seasons in women, with higher levels in spring/summer than autumn/winter, providing evidence for seasonal variation in serotonergic gene regulation.

## INTRODUCTION

As the enzyme primarily responsible for degradation of serotonin, monoamine oxidase A (MAO-A) is integral to monoaminergic homeostasis in the human brain. Alterations to MAO-A function have been associated with risk (Ducci et al., 2008; Weder et al., 2009), pathophysiology, and treatment (Chiuccariello et al., 2015) of various psychiatric illnesses. These include affective (Meyer et al., 2006), anxiety (Reif et al., 2014), obsessive compulsive (Taylor, 2013), substance use (Matthews et al., 2014), and personality disorders (Kolla et al., 2016). In depression, MAO-A hyperactivity is thought to result in reduced serotonin signaling (Meyer et al., 2006). Information provided by peripheral assessments of MAO function is limited, as platelet MAO expression is restricted to that of MAO-B, while the brain expresses both isoenzymes (Shih et al., 1999). Positron emission tomography (PET) with [^11^C]harmine provides specific in vivo information on brain MAO-A density and distribution (Bergstrom et al., 1997a; Ginovart et al., 2006). In particular, [^11^C]harmine PET studies have provided evidence for changes to MAO-A distribution volume (V_T_), an index of protein levels, in major depression (Meyer et al., 2006).

Changes to DNA methylation within the *MAOA* gene have been observed in multiple psychiatric conditions (Domschke et al., 2012; Peng et al., 2018; Schiele et al., 2018, 2020; Ziegler and Domschke, 2018; Ziegler et al., 2018). They are mediated both by risk factors for (Checknita et al., 2018, 2021)—as well as treatment of (Ziegler et al., 2016; Schiele et al., 2018)—these diseases, suggestive of a role of *MAOA* gene methylation as an intermediary between environment and neurobiology. In vitro studies demonstrate a negative association between peripheral blood *MAOA* promoter/intron I/exon I methylation and protein function (Checknita et al., 2015; Schiele et al., 2018). Methylation of this region has been linked to clinical conditions (Melas and Forsell, 2015; Bendre et al., 2018; Checknita et al., 2018). In theory, methylation of this region may be associated with particularly strong downregulation of transcription (Brenet et al., 2011). In a PET study utilizing in vivo [^11^C]clorgyline in healthy individuals, a negative association between *MAOA* promoter methylation and brain MAO levels was demonstrated (Shumay et al., 2012). However, the effect of promoter/exon I/intron I methylation on human in vivo brain MAO-A levels in patients with depression, as measured by [^11^C]harmine, has yet to be assessed.

Seasonal affective disorder (SAD) is characterized by depressive symptoms in autumn and winter and remission in spring and summer. Though evidence points toward a serotonergic pathophysiology (Neumeister et al., 2000; Praschak-Rieder et al., 2008) and efficacy of MAO inhibitors such as moclobemide are suggestive of a role for MAO-A (Lingjaerde et al., 1993), a study by our group did not find altered MAO-A V_T_ in SAD (Spies et al., 2018). However, the role of epigenetic mechanisms in driving differences in cerebral MAO-A levels is insufficiently understood.

Here we assessed (1) the effect of average and CpG-specific *MAOA* promoter/exon I/intron I region DNA methylation on brain MAO-A V_T_ assessed with [^11^C]harmine PET in 30 healthy individuals and 22 patients with winter-type SAD. (2) We additionally took the seasonal pathophysiology of SAD into consideration by probing the impact of season on *MAOA* DNA methylation.

## METHODS

### Study Design

The current study utilizes [^11^C]harmine PET and *MAOA* DNA methylation data from 30 healthy controls (HCs) and 22 patients with winter-type SAD (n = 52). Data were gleaned from a previously published study assessing changes to MAO-A V_T_ in SAD after treatment with bright light therapy (BLT) and across the seasons (Spies et al., 2018) that comprised a screening visit, 3 PET measurements (PET1, before treatment in autumn/winter; PET2, after treatment in autumn/winter; and PET3, after treatment in spring/summer), a structural magnetic resonance imaging scan, and a follow-up visit. A blood draw for methylation analysis was performed at either PET1, PET3, or the follow-up visit. Here, we utilized the PET scan (PET1 or PET3) that was performed closest to genetic/epigenetic blood sampling (mean difference 27.63 ± SD, 51.74 days between PET and blood draw). At PET3, patients were remitted from SAD, and some individuals had received BLT or placebo. The study was conducted in accordance with the Declaration of Helsinki, including all current revisions and the good scientific practice guidelines of the Medical University of Vienna. The protocol was approved by the ethics committee of the Medical University Vienna (EK Nr.: 1681/2016) and registered at clinicaltrials.gov (NCT02582398).

### Participants

SAD patients were recruited via the respective outpatient clinic at the Department of Psychiatry and Psychotherapy, Medical University of Vienna. HCs were recruited via advertisements in local newspapers, electronic media, and dedicated message boards at the Medical University of Vienna. The Structured Clinical Interview for DSM-IV Axis I disorders was used to diagnose unipolar, winter-type SAD and exclude psychiatric comorbidities in patients as well as to confirm psychiatric health in HCs. In addition, to confirm (SAD patients) or exclude (HCs) the diagnosis of SAD, all individuals completed the Seasonal Pattern Assessment Questionnaire (Raheja et al., 1996). Participants were free from psychopharmacologic medication for the period of study participation and within 6 months prior to study enrollment. Severe somatic illness, neurologic comorbidities, current drug abuse, current smoking, and pregnancy (female participants) were excluded based on medical history, routine laboratory parameters (blood draw and urine tests), electrocardiography, and physical examination performed at the screening visit. All individuals provided written informed consent and received financial reimbursement for their participation.

### Positron Emission Tomography

PET scans were performed with a GE Advance full-ring PET scanner (GE Medical Systems, Waukesha, WI, USA) at the Department of Biomedical Imaging and Image-guided Therapy, Medical University of Vienna. [^11^C]harmine (7-[^11^C]methoxy-1-methyl-9H-[3,4-b]indole) synthesis and quality control were performed in line with the workflow presented by (Philippe et al., 2015). In a first step, a 5-minute transmission scan was performed with ^68^GE rod sources for tissue attenuation. Dynamic PET scans started simultaneously with the i.v. bolus application of [^11^C]harmine (4.6 MBq/kg body weight) (Bergstrom et al., 1997b; Ginovart et al., 2006). All scans were acquired in 3D mode, collecting 51 successive time frames (12 × 5 seconds, 6 × 10 seconds, 3 × 20 seconds, 6 × 30 seconds, 9 × 1 minute, and 15 frames × 5 minutes), resulting in a total acquisition time of 90 minutes. Scans were reconstructed into 35 transaxial section volumes (128 × 128 matrix) utilizing an iterative filtered back-projection algorithm (FORE-ITER) with a spatial resolution of 4.36 mm full width at half maximum 1 cm next to the center of the field of view. Additionally, arterial blood samples for [^11^C]harmine quantification were drawn via automated blood sampling for the first 10 minutes of measurement (ALLOGG, Mariefred, Sweden), complemented by manual sampling at 5, 10, 20, 30, 45, 60, and 80 minutes after tracer application.

### Magnetic Resonance Imaging

T1-weighted MR images (magnetization prepared rapid gradient echo sequence, 256 × 240 matrix, 1 × 1 mm voxel size, slice thickness 1.1 mm, 200 slices) were acquired using a 3 Tesla PRISMA MR Scanner (Siemens Medical, Erlangen, Germany) at the Medical University of Vienna.

### MAO-A V**_T_ Quantification**

Prior to quantification, each PET scan was spatially normalized to Montreal Neurological Institute (MNI) space using SPM12 (Wellcome Trust Centre for Neuroimaging, London, UK; http://www.fil.ion.ucl.ac.uk/spm/). In short, each PET was corrected for head motion and co-registered to the T1 structural image. Afterwards, each MR scan was normalized to MNI space utilizing a tissue probability map, producing the transformation matrix that was used to normalize the co-registered PET scan to MNI space.

Manually drawn arterial blood samples were processed according to the protocol published by (Ginovart et al., 2006). A gamma counter was cross-calibrated with the PET scanner as well as the automated arterial blood sampling system. Arterial blood samples drawn at 5 and 10 minutes were used for cross-calibration between the gamma counter and PET scanner. To acquire non-metabolized [^11^C]harmine in arterial blood as a function of time, the arterial input function was calculated as the product of whole blood activity (fit with 3 exponentials), plasma-to-whole blood ratio (linear fit), and the fraction of non-metabolized tracer concentration in arterial plasma (fit with Watabe function).

Logan plot was used for voxel-wise quantification of MAO-A V_T_. Previous studies have shown that quantification of MAO-A V_T_ using the logan plot is stable and comparable with compartment modeling strategies (Ginovart et al., 2006; Spies et al., 2018). Thereby, the estimated arterial input function and the time activity curve of thalamus, representing the high uptake region, were used. Regional V_T_ were extracted for frontal and temporal pole, anterior and posterior cingulate gyrus, thalamus, caudate, putamen, hippocampus, and midbrain as adopted from the Harvard Oxford Structural atlas (http://fsl.fmrib.ox.ac.uk/fsl/fslwiki/Atlases) as well as striatum taken from an in-house atlas (Savli et al., 2012). Afterwards, a global region of interest (ROI) representing the weighted average of regional V_T_ was utilized, as relevant regional differences in methylation effects on MAO-A V_T_ were not hypothesized and because MAO-A V_T_ was highly correlated between regions (average correlation = 0.92 ± 0.04). PMOD 3.509 (PMOD Technologies Ltd., Zurich, Switzerland; www.pmod.com) was used to fit the arterial input function and for [^11^C]harmine quantification.

### DNA Sampling and Isolation

Venous blood (approximately 24 mL) was collected in ethylenediaminetetraacetic acid (EDTA) blood tubes and stored at −80°C. DNA extraction was performed using the QIAamp DNA Blood Midi and Maxi Kit (Qiagen, Hilden, Germany) according to the manufacturer’s recommendations at the Department of Psychiatry, Psychotherapy and Psychosomatics of the University of Halle, Germany. Afterwards, DNA samples were again stored at −80°C.

### *MAOA* VNTR Genotyping and Methylation Analysis

Participants were genotyped for the *MAOA* variable number of tandem repeat (VNTR) promoter polymorphism containing 2, 3, 3.5, 4, or 5 copies of the repeated sequence, as this variant was shown in vitro to affect MAO-A expression (Sabol et al., 1998) and was related to depression (Du et al., 2002; Gutiérrez et al., 2004; Yu et al., 2005). *MAOA* VNTR genotyping was performed at the Department of Psychiatry, Psychotherapy and Psychosomatics of the University of Halle, Germany. Briefly, 25-µL PCR reactions containing 50 ng DNA, 10 pMol each of forward (5’-TGCTCCAGAAACATGAGCAC-3’) and reverse primers (5’- ATTGGGGAGTGTATGCTGGA-3‘), 1 U Taq polymerase, 10 mmol dNTPs, 15 mM ammonium sulfate, 60 mM Tris-HCl (pH 9.5), and 1.5 mmol/µL MgCl_2_ were amplified in 35 cycles (94°C for 30 seconds; 56°C for 30 seconds; 72°C for 1 minute) after an initial denaturation step at 94°C for 5 minutes and PCR fragments were resolved on a 2.5% agarose gel.

DNA methylation was then assessed at the Department of Psychiatry and Psychotherapy, University of Freiburg, Faculty of Medicine, Germany, via direct sequencing of bisulfite-converted DNA. Degree of methylation at 13 CpG sites located in an amplicon comprising promoter/exon I/intron I of *MAOA* (chromosome X, GRCh38.p2 Primary Assembly, NCBI Reference Sequence: NC_000023.11, 43656260–43656613) was analyzed individually. These CpG sites were numbered in accordance with prior studies on *MAOA* methylation in psychiatric diseases: CpG 1 = 43 656 316; CpG 2 = 43 656 327; CpG 3 = 43 656 362; CpG 4 = 43 656 368; CpG 5 = 43 656 370; CpG 6 = 43 656 383; CpG 7 = 43 656 386; CpG 8 = 43 656 392; CpG 9 = 43 656 398; CpG 10 = 43 656 427; CpG 11 = 43 656 432; CpG 12 = 43 656 514; CpG 13 = 43 656 553 (Domschke, 2012; Ziegler et al., 2018). For detailed information on *MAOA* methylation analysis, see (Ziegler et al., 2016). Average methylation of these 13 CpG sites was determined.

### Statistical Analysis

Statistical tests were performed using SPSS version 28 for Windows (SPSS Inc., Chicago, IL, USA).

#### Analysis of MAOA DNA Methylation

**—** *MAOA* methylation data were available for 29 SAD patients (mean age, 32.83 ± SD 9.55 years, 18 females) and 44 HCs (mean age, 34.27 ± SD 9.86 years, 26 females). Average *MAOA* methylation was defined as the average methylation of all pre-defined CpG sites (CpG 1-13).

Based on Shapiro–Wilk tests for normality and visual inspection, non-parametric testing was used with average *MAOA* methylation as the primary outcome parameter. Mann-Whitney U test was used to probe for differences in average *MAOA* methylation between males and females, SAD patients and HCs, the effect of season (methylation analysis in spring/summer, covering the period from April to September vs autumn/winter, covering the period between October and March), and of VNTR high-expressing (3.5 and 4 repeats) vs low-expressing group (2, 3, and 5 repeats) (Sabol et al., 1998).

Inter-correlation of CpG sites ranged from −0.22 to 0.86 in females and from −0.24 to 0.77 in males (see supplementary Figures 1–3). Thus, exploratory analyses of the effects of age, sex, group (SAD patients vs healthy controls), season, and VNTR genotype on individual CpG site methylation levels were performed utilizing the same steps as for average methylation.

#### Effect of *MAOA* DNA Methylation on MAO-A VT

**—**PET data were available for 22 SAD patients (mean age, 33 ± 10.20 years, 14 females) and 30 HCs (mean age, 33.80 ± 9.76 years, 17 females). A general linear model (GLM) was utilized using global MAO-A V_T_ as the dependent variable. Sex, VNTR expression group, season, and group (SAD patients vs HCs) were used as fixed factors and age (Z-scored) as covariate to probe for their potential effect on MAO-A V_T_ for the subsequent analysis.

The effect of average *MAOA* methylation on global MAO-A V_T_ was assessed using GLM, with global MAO-A V_T_ as the dependent variable and average methylation (Z-scored) as the covariate. Based on previous evidence for an effect of sex, season, and depression on brain MAO-A V_T_ (Meyer et al., 2009; Rekkas et al., 2014; Spies et al., 2018), a second GLM was computed additionally including sex, group (SAD patients vs HCs), and season as fixed factors.

Furthermore, an independent sample *t* test was conducted comparing MAO-A V_T_ between SAD patients and HCs in individual ROIs (frontal pole, temporal pole, ACC, PCC, thalamus, caudate, putamen, hippocampus, midbrain, and striatum).

In an exploratory manner, GLM analysis was repeated to assess the effects of individual CpG cite methylation on global MAO-A V_T_. Also, GLM analysis was repeated to assess the effects of average methylation on MAO-A V_T_ within the individual ROIs (frontal pole, temporal pole, ACC, PCC, thalamus, caudate, putamen, hippocampus, midbrain, and striatum) included in the global ROI, based on studies suggesting that PET may underestimate differences between regions (Tong et al., 2013).

Multiple testing was corrected using the Holm-Bonferroni method and significance was set at *P* < .05.

## RESULTS

### Analysis of *MAOA* DNA Methylation

**Figure 1.**
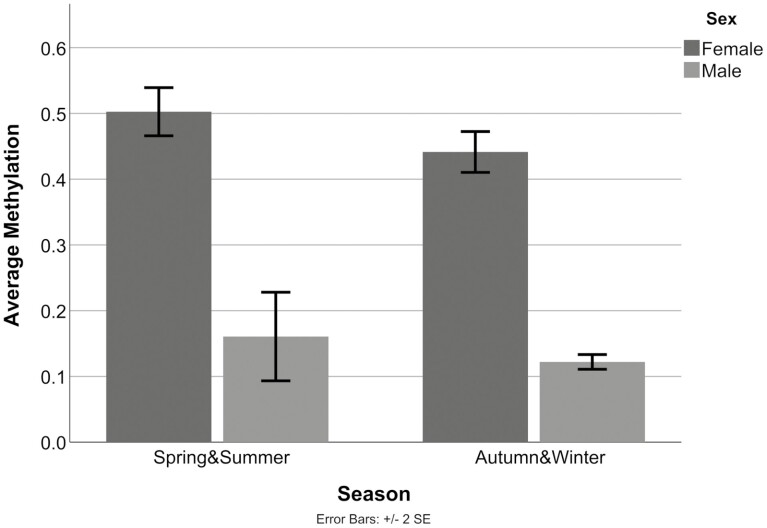
Lower average *MAOA* promoter/exon I/intron I region DNA methylation in autumn and winter compared with spring and summer within the female participant group (all women, 2-sided Mann–Whitney U test, *P*_uncorr = _.030).

Mann–Whitney U test revealed a significant effect of sex on average methylation, with higher levels in females (*P*_corr_ < .05), which was expected based on the X-chromosomal location of *MAOA*. Hereafter, methylation data of male and female participants were investigated separately. No effect of group (SAD patients vs HCs) or VNTR genotype on average methylation was detected. As shown in Figure 1, an effect of season on average methylation was observed in female participants (all women, i.e., healthy women and women suffering from SAD), showing higher methylation levels in women whose blood was drawn during spring or summer vs autumn or winter (2-sided Mann–Whitney U test, *P*_uncorr_ = .03). This finding did not survive correction for multiple comparisons.

Results of exploratory analyses of the effects of age, sex, group (SAD patients vs HCs), season, and VNTR genotype on individual CpG site methylation levels are presented in the supplement. Mean methylation levels are presented in the supplement as well (supplementary Tables 1 and 2).

### Effect of *MAOA* DNA Methylation on MAO-A V_**T**_

Initial GLM implemented to detect potential covariates (excluding methylation data) revealed no significant effect of group (SAD patients vs HCs), season, or VNTR genotype on MAO-A V_T_. However, as shown in Table 1, an effect of sex on MAO-A V_T_ could be found, with higher MAO-A V_T_ in females compared with males (*P*_uncorr_ = .03).

**Table 1.** Effect of potential covariates on global MAO-A V_T_

Variable	Value label	N	Mean ± SD MAO-A V_T_	*P*
Sex	Male	21	13.74 ± 3.22	.03*
	Female	31	14.90 ± 3.18	
Season	Spring/summer	24	14.04 ± 3.05	.11
	Autumn/winter	28	14.76 ± 3.42	
Health status	PAT	22	14.11 ± 3.69	.41
	HC	30	14.66 ± 2.87	
uVNTR	High	34	14.84 ± 3.42	.12
	Low	18	13.65 ± 2.71	

Abbreviations: df, degrees of freedom; GLM, general linear model; HC, healthy controls; MAO-A V_T_, monoamine oxidase A distribution value; PAT, patients; uVNTR, upstream variable number of tandem repeats. GLM implemented to detect potential covariates (i.e., health status, season or VNTR genotype) on global MAO-A V_T_ (* = significant at *P*_uncorr_ < .05).

Average *MAOA* DNA methylation had no significant effect on global MAO-A V_T_, regardless of whether group (SAD patients vs HCs), sex, or season were corrected for via inclusion as covariates (*P*_uncorr_ = .20) or not (*P*_uncorr_ = .68).

In addition, no significant differences in global or ROI-specific MAO-A V_T_ between SAD patients and HCs was detected. Average global and ROI-specific MAO-A V_T_ for both SAD patients and HCs are summarized in Table 2.

**Table 2.** MAO-A V_T_ between SAD patients and HC in individual ROIs

ROI	Health status	Mean ± SD MAO-A V_T_	*P*
Global ROI	HC	14.66 ± 2.87	.89
	PAT	14.11 ± 3.69	
Frontal	HC	13.99 ± 2.82	.07
	PAT	13.39 ± 3.69	
Temporal	HC	13.56 ± 2.67	.82
	PAT	13.59 ± 3.27	
ACC	HC	16.40 ± 3.21	.82
	PAT	15.75 ± 4.20	
PCC	HC	16.39 ± 3.19	.88
	PAT	15.62 ± 3.84	
Thalamus	HC	18.84 ± 3.95	.82
	PAT	17.76 ± 4.85	
Caudate	HC	11.66 ± 2.66	.96
	PAT	11.48 ± 3.13	
Putamen	HC	16.01 ± 3.02	.78
	PAT	15.52 ± 4.17	
Hippocampus	HC	15.85 ± 3.15	1.00
	PAT	15.07 ± 3.84	
Midbrain	HC	18.72 ± 3.78	.60
	PAT	18.04 ± 4.21	
Striatum	HC	10.45 ± 2.31	.78
	PAT	9.84 ± 2.58	

Abbreviations: ACC, anterior cingulate cortex; HC, healthy controls; MAO-A V_T_, monoamine oxidase A distribution value; PAT, patients; PCC, posterior cingulate cortex; ROI, region of interest; SAD, seasonal affective disorder. Results of the independent sample *t* test comparing MAO-A V_T_ between SAD patients (n = 22) and HCs (n = 30) in individual ROIs (* = significant at *P*_uncorr_ < .05).

Results from exploratory analyses of specific CpG site methylation on global MAO-A V_T_ and average *MAOA* methylation on specific ROIs are reported in the supplement.

## DISCUSSION

Here, we explored the effect of *MAOA* DNA methylation on global brain MAO-A V_T_ in HCs and patients with SAD. In contrast to prior findings on the effect of *MAOA* promoter methylation on cerebral MAO-A levels (Shumay et al., 2012), no statistically significant association was observed for average methylation of the 13 CpG sites assessed here (promoter/exon I/ intron I). In addition to well-known sex differences in methylation of X-chromosomal genes (Nugent and McCarthy, 2011) such as *MAOA*, we detected a statistically significant effect (uncorrected) of season on *MAOA* DNA methylation, with higher methylation levels in spring/summer. Group (SAD patients vs HCs) and *MAOA* promoter VNTR genotype (grouped by high-/low-expressing alleles) did not affect average *MAOA* DNA methylation.

### Analysis of *MAOA* DNA Methylation

We observed higher *MAOA* methylation in samples that were collected during spring or summer in women. Although this association did not survive correction for multiple comparisons, it is in accordance with the role of season within the serotonergic system (Mc Mahon et al., 2016) and suggests increased serotonin availability during spring/summer as conferred by lower MAO-A activity due to *MAOA* hypermethylation. Restriction of this effect to the female sex is potentially related to the aforementioned X-linked nature of *MAOA* and general consequences for methylation (Lyon, 1961; Pinsonneault et al., 2006; Nugent and McCarthy, 2011; Domschke et al., 2015).

In general, epigenetic adaptations such as DNA methylation are known to be sensitive to a variety of external influences (Lam et al., 2012) such as drugs (Webb et al., 2020), exposure to environmental stimuli (Tao et al., 2014; Martin and Fry, 2018), toxins (Lambrou et al., 2012; Kim et al., 2013; Hernandez-Vargas et al., 2015), tobacco smoke (Launay et al., 2009; Shenker et al., 2013), or nutritional factors (Crider et al., 2012). Furthermore, Bind and colleagues observed that environmental temperature and relative humidity were associated with dynamic changes in DNA methylation (Bind et al., 2014b). In line with this assumption, Ricceri et al. reported higher mean methylation in spring and summer for certain genes (Ricceri et al., 2014). Notably, such findings are not limited to humans, with changes to methylation observed in hibernating animals (Fujii et al., 2006; Alvarado et al., 2014). Thus, some environmental, nutritional, and biologic factors that follow a seasonal pattern affect methylation (Scoccianti et al., 2011; Park et al., 2012; Azzi et al., 2014; Bind et al., 2014a). Moreover, expression of various proteins within the serotonergic system was shown to be sensitive to season and light (Praschak-Rieder et al., 2008; Spindelegger et al., 2012; Harrison et al., 2015; Matheson et al., 2015; Tyrer et al., 2016). Based on our methylation results, one might hypothesize that epigenetic processes facilitate these seasonal protein-level changes. However, seasonal effects on methylation did not carry over to changes in MAO-A V_T_ expression in our study, suggesting that these effects are minor compared with other variables (Bacher et al., 2011) or pathologic conditions known to affect MAO-V_T_ (Meyer et al., 2009).

Again, we did not detect evidence for a significant effect of SAD on average *MAOA* DNA methylation. As summarized by (Ziegler and Domschke, 2018), changes to average *MAOA* methylation of the genetic subregion we assessed were shown in psychiatric conditions (Melas et al., 2013; Melas and Forsell, 2015). Furthermore, Peng et al. investigated changes in *MAOA* promoter region methylation in depressive states, showing that methylation was negatively associated with depressive symptoms (Peng et al., 2018). Thus, in conjunction with this literature, our study is suggestive of a different or lesser role of *MAOA* methylation in SAD pathophysiology than is the case for non-seasonal depression. On a theoretical level, this is in accordance with authors who promote the concept of SAD as an individual disease entity (Bauer and Dunner, 1993).

Finally, we did not find an effect of *MAOA* VNTR genotype on average *MAOA* DNA methylation of the amplicon comprising promoter/exon I/intron I. VNTR was taken into consideration based on its substantial effect on MAO-A function (Sabol et al., 1998) and thus potential impact on MAO-A V_T_, methylation, or both. An association of VNTR genotype and *MAOA* DNA methylation levels has been discussed (Philibert et al., 2008), though contradicted by others (Shumay et al., 2012). It should be considered that, compared with the presently investigated promoter/exon I/intron I region, the VNTR sequence is located further 5’ within the promoter of *MAOA.* Thus, any effects would be indirect, for example via secondary effects on other regulatory processes that include the promoter.

### Effect of *MAOA* DNA Methylation on MAO-A V_**T**_

One previous study assessed the effect of peripheral *MAOA* methylation on brain MAO-A levels using [^11^C]clorgyline (Shumay et al., 2012). The authors reported an association between CpG site specific *MAOA* core promoter methylation further 5’ and brain MAO-A levels in healthy individuals. Here, we assessed methylation within a promoter/exon I/intron I region, based on previous literature demonstrating altered methylation of this sequence in psychiatric disorders (Ziegler et al., 2016, 2018) and prior observations that exon I methylation in general may result in particularly strong downregulation of transcription (Brenet et al., 2011). As increased MAO-A is understood as an endophenotype of affective disorders (Meyer et al., 2006), we postulated that altered DNA methylation may facilitate changes in MAO-A V_T_ previously observed in depression. The lack of an association in our study may thus be related to the methylation sites we assessed and could be suggestive of a stronger association between promoter methylation and cerebral MAO-A. Furthermore, we did not find a significant difference between SAD and HC in V_T_ in this sample or in an overlapping sample previously published (Spies et al., 2018), highlighting that, even if *MAOA* methylation did have an effect of VT, this would be of limited pathophysiologic relevance.

However, the choice of tracer may also underlie the observed differences in findings between studies. These involve differences between [^11^C]clorgyline and [^11^C]harmine in enzyme-binding specificity and kinetics, with [^11^C]harmine showing markedly higher MAO-A specificity (Bergström et al., 1997a). Moreover, in contrast to [^11^C]harmine, [^11^C]clorgyline binds irreversibly to MAO (Fowler et al., 1987) and exhibits characteristics that may limit data quality, including the presence of radioactive metabolites with MAO affinity (Narayanaswami et al., 2019). Importantly, [^11^C]harmine has developed as the most commonly utilized radioligand for brain MAO-A imaging in psychiatry (Meyer et al., 2006, 2009; Spies et al., 2018; Baldinger-Melich et al., 2019). Therefore, further investigations with a comparable study design are needed to elucidate the role of *MAOA* DNA region in the relationship between methylation and cerebral MAO-A. Moreover, a wide range of post-transcriptional processes regulate serotonergic protein expression, as illustrated by recent studies demonstrating only weak or no association between mRNA and protein levels (Komorowski et al., 2017; Murgaš et al., 2022; Godbersen et al., 2022). Importantly, Komorowski et al. found a significant link between gene and protein expression for certain serotonin receptors (5-HT_1A_ and 5-HT_2A_). In contrast, only a weak association was shown for MAO-A when PET binding values and gene as well as protein expression were correlated (Komorowski et al., 2017). These factors, which cannot be addressed within our study design, may obscure the effects of methylation on cerebral MAO-A V_T_.

### Limitations

Analysis of *MAOA* DNA methylation requires a sex-specific approach, which results in smaller sample sizes. Because methylation is susceptible to a wide variety of influences (Lam et al., 2012; Martin and Fry, 2018), variation is high, potentially obscuring smaller effect sizes. As a result, many studies of x-linked genes limit testing to male participants (Shumay et al., 2012). However, assessment in both sexes is paramount for a more comprehensive understanding of x-linked genetic and epigenetic processes. Our study sample size, though comparable with that of other MAO-A PET studies (Meyer et al., 2006; Attwells et al., 2017; Moriguchi et al., 2019; Svensson et al., 2021; Godbersen et al., 2022), in particular other studies addressing the impact of methylation (Shumay et al., 2012)_,_ can be considered relatively small. However, our study is adequately powered to detect effect sizes on the lower end of medium (f^2^ = 0.156) (Selya et al., 2012) with an alpha = .05, power = 0.8. Thus, our non-significant results demonstrate that average *MAOA* promoter/exon I/intron I region DNA methylation has, if any, only small effects on MAO-A V_T_. The study at hand assesses methylation of a DNA region (*MAOA* promoter/exon I/intron I) located further 3’ than that used in the study performed by (Shumay et al., 2012). This decision was hypothesis driven based on the region’s clinical implications and potentially potent effect on protein expression (Domschke et al., 2012; Ziegler et al., 2016, 2018; Ziegler and Domschke, 2018). However, it limits comparability to prior findings that focus on the *MAOA* promoter (Shumay et al., 2012). To utilize PET data with temporal proximity to the time point of methylation analysis, PET data acquired in spring/summer were used in some participants. Thereby, some SAD patients were remitted at the time of assessment and some had received either BLT or placebo. Thus, for some individuals, sustained effects of BLT on *MAOA* DNA methylation or MAO-A expression cannot be ruled out.

### Conclusion

Here we aimed to assess the effect of *MAOA* promoter/exon I/intron I region DNA methylation (Ziegler et al., 2016, 2018) on cerebral MAO-A V_T_ assessed with [^11^C]harmine PET. We also probed the influence of SAD, season, and *MAOA* VNTR genotype. We did not find evidence for an effect of *MAOA* promoter/exon I/intron I region DNA methylation on brain MAO-A V_T_, in contrast to a previous PET study that demonstrated an association between *MAOA* promoter methylation and brain MAO-A levels. Thus, compared with a region within the promoter located further 5’, *MAOA* promoter/exon I/intron I region DNA methylation only appears to have limited, if any, impact on brain protein levels. Importantly, the use of different radiotracers must be considered. We observed an effect of season on average methylation in females irrespective of their clinical health status (i.e., both in women suffering from SAD and in healthy women), which is in accordance with evidence for seasonal changes within the serotonergic system.

## Supplementary Material

pyac085_suppl_Supplementary_Material

## Data Availability

The data underlying this article cannot be shared publicly due to ethical reasons. The data will be shared on reasonable request to the corresponding author according to the guidelines of—and after consulting with appropriate representatives of—the primary affiliation (Medical University of Vienna, AT).

## Interest Statement

R. Lanzenberger received investigator-initiated research funding from Siemens Healthcare regarding clinical research using PET/MR. He is a shareholder of the start-up company BM Health GmbH since 2019. D. Winkler received lecture fees/authorship honoraria from Angelini, Lundbeck, Medical Dialogue, and MedMedia Verlag. P. Handschuh received authorship honoraria from MedMedia Verlag and a travel grant from Angelini. K. Domschke is a member of the Steering Committee Neurosciences, Janssen Inc. M. Spies received speaker honoraria from Janssen and Austroplant as well as travel grants and/or workshop participation from Janssen, Austroplant, AOP Orphan Pharmaceuticals, and Eli Lilly. D. Rujescu served as consultant for Janssen, received honoraria from Gerot Lannacher, Janssen and Pharmagenetix, received travel support from Angelini and Janssen, and served on advisory boards of AC Immune, Roche and Rovi. The other author(s) declared no potential conflicts of interest with respect to the research, authorship, and/or publication of this article.
