# Supplementary material for: Effect of MAOA DNA Methylation on Human in Vivo Protein Expression Measured by [11C]harmine Positron Emission Tomography
Source: Int J Neuropsychopharmacol. 2022 Dec 27;26(2):116–24. doi: 10.1093/ijnp/pyac085 (PMC9926052; doi:10.1093/ijnp/pyac085)
Supplement: pyac085_suppl_Supplementary_Material [file pyac085_suppl_supplementary_material.docx]

***Supplement***

**Effect of *MAOA* DNA methylation on human *in vivo* protein expression measured by [^11^C]harmine positron emission tomography**

*MAOA DNA methylation and [^11^C]harmine PET*

Patricia A. Handschuh^1^, Matej Murgaš^1^, Chrysoula Vraka^2^, Lukas Nics^2^,

Annette M. Hartmann^1^, Edda Winkler-Pjrek^1^, Pia Baldinger-Melich^1^, Wolfgang Wadsak^2,3^, Dietmar Winkler^1^, Marcus Hacker^2^, Dan Rujescu^1^, Katharina Domschke^4,5^, Rupert Lanzenberger^1^*, Marie Spies^1^

*^1^Department of Psychiatry and Psychotherapy, Medical University of Vienna*

*^2^Department of Biomedical Imaging and Image-guided Therapy, Division of Nuclear Medicine, Medical University of Vienna*

*^3^Center for Biomarker Research in Medicine (CBmed), Graz, Austria*

*^4^Department of Psychiatry and Psychotherapy, Medical Center - University of Freiburg, Faculty of Medicine, Freiburg, Germany*

*^5^Centre for Basics in Neuromodulation, Faculty of Medicine, University of Freiburg, Germany*

*Corresponding Author:

Prof. Rupert Lanzenberger, MD, PD

Department of Psychiatry and Psychotherapy, Medical University of Vienna, Austria

[rupert.lanzenberger@meduniwien.ac.at](mailto:rupert.lanzenberger@meduniwien.ac.at)

T: +43 1 40400 35760

F: +43 1 40400 30990

<http://www.meduniwien.ac.at/neuroimaging/>

Clinicaltrials.gov Identifier: NCT02582398

(<https://clinicaltrials.gov/ct2/show/NCT02582398>)

EUDAMED Number: CIV-AT-13-01-009583

For submission to:

***International Journal of Neuropsychopharmacology***

**Exploratory analyses**

**Results**

**Analysis of MAOA DNA methylation**

In exploratory analyses of individual CpGs, higher methylation at CpG 1 (p_uncorr_ = <0.01) and at CpG 3 (p_uncorr_ <0.01) in healthy females compared to females with SAD was observed. In males, those suffering from SAD showed higher methylation at CpG 5 (p_uncorr_ = 0.03) than healthy controls. Additionally, significantly higher methylation was found at CpGs 2 (p_uncorr_ = <0.01), 3 (p_uncorr_ = 0.04), 5 (p_uncorr_ < 0.05), 7 (p_uncorr_ = 0.04) and 9 (p_uncorr_ = 0.02) in samples of females that were collected during spring or summer compared to those collected in autumn or winter. See *Table S1* and *S2* for mean methylation levels.

**Effect of MAOA DNA methylation on MAO-A VT**

Exploratory analyses of specific CpG site methylation on global MAO-A V_T_ did not reveal significant effects. Analyses of ROI-specific (i.e. frontal pole, temporal pole, ACC, PCC, thalamus, caudate, putamen, hippocampus, midbrain, striatum) MAO-A V_T_ revealed no effects of average *MAOA* DNA methylation.

**Discussion**

Average methylation was utilized as a primary outcome parameter in accordance with previous clinical studies. However, to address middle to low inter-correlation between methylation of specific, particularly outer CpGs (see *Figure S1*), CpG site specific analysis was also performed ([Domschke et al., 2015](#_ENREF_1)). These exploratory analyses demonstrated an effect of health status (patients vs. controls) on methylation levels of individual *MAOA* CpG sites in each sex as well as an effect of season in females. Thus, these findings are partially in accordance with the effect of season on average methylation and point towards potential changes within SAD. However, despite prior findings on the pathophysiological relevance of the pre-defined *MAOA* CpG sites in our sample ([Ziegler et al., 2016](#_ENREF_2); [Ziegler et al., 2018](#_ENREF_3)), the number of tests CpG-specific assessments require necessitates investigations in a larger sample.

**Supplementary Figures**


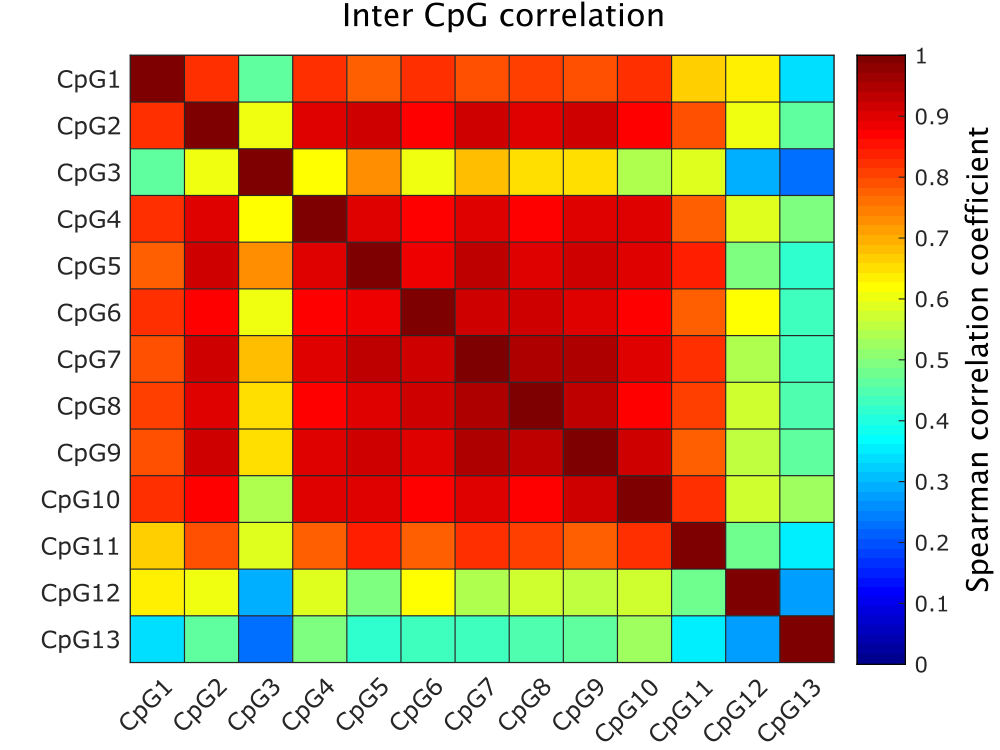


Figure S1: Correlation of mean methylation across all subjects between individual CpGs. The color chart represents spearman correlation coefficients.


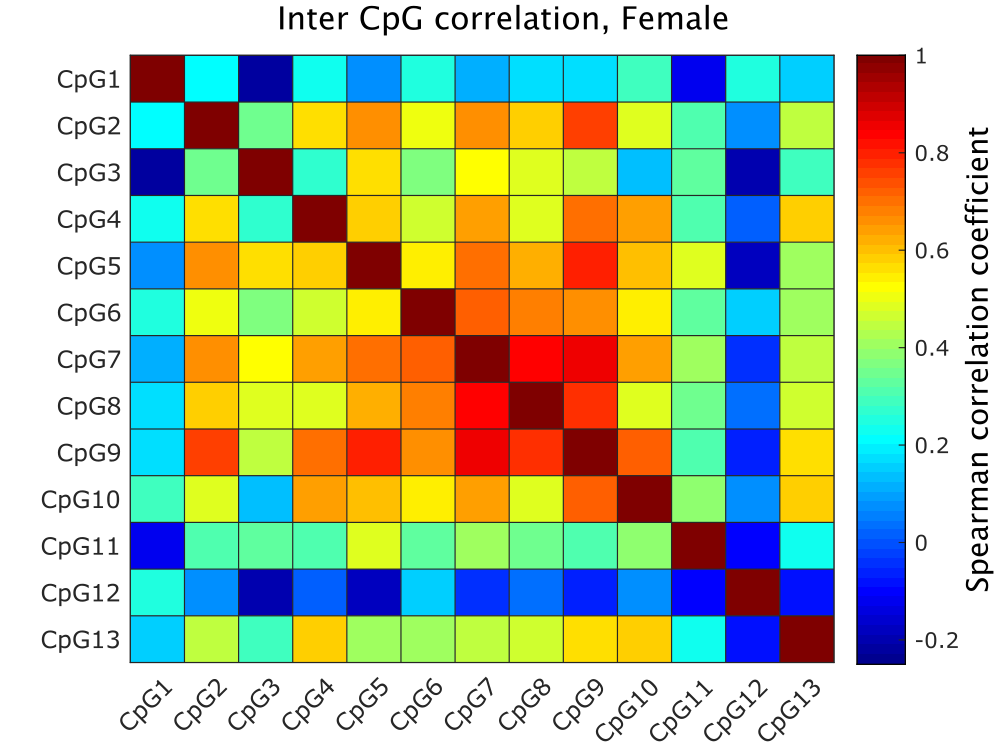


Figure S2: Correlation of mean methylation across female subjects between individual CpGs. The color chart represents spearman correlation coefficients.


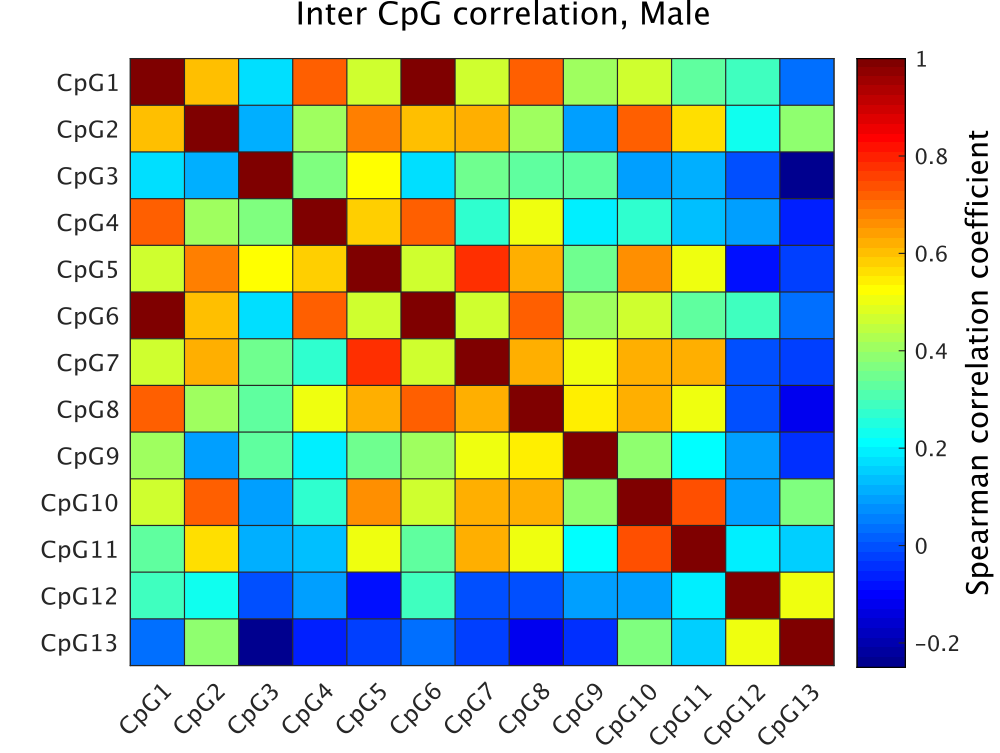


Figure S3: Correlation of mean methylation across male subjects between individual CpGs. The color chart represents spearman correlation coefficients.


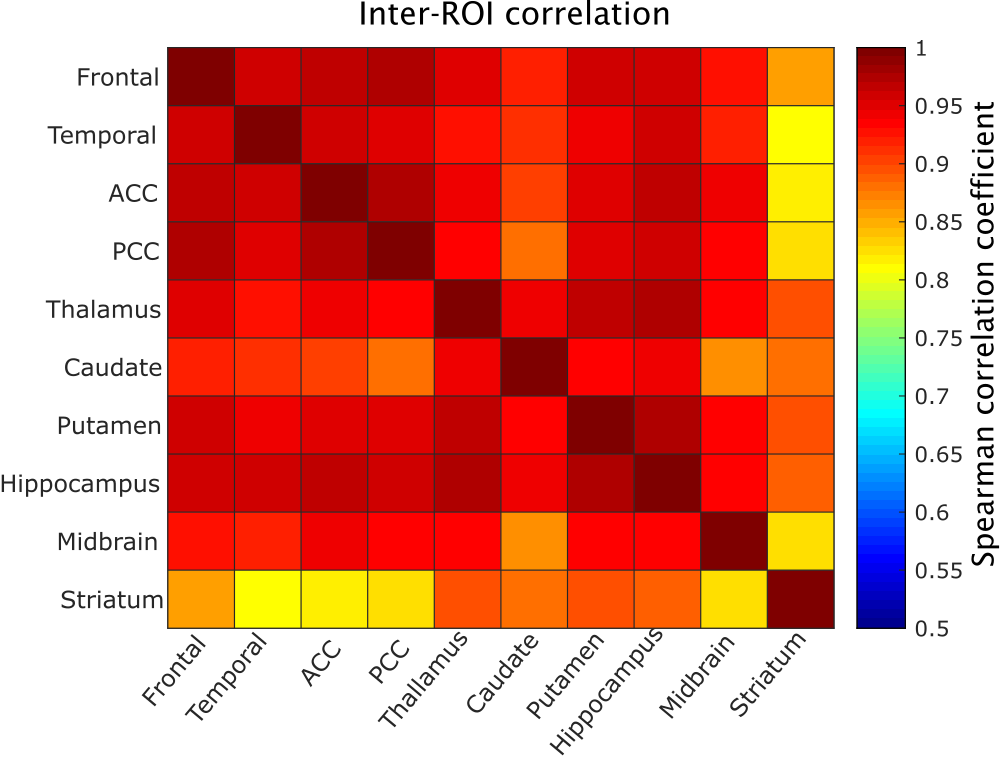


Figure S4: Correlation of mean monoamine oxidase A distribution volume (MAO-A V_T_) across subjects between individual regions of interest (ROIs). Color chart represents spearman correlation coefficients.

**References**

Domschke K, Tidow N, Schwarte K, Ziegler C, Lesch K-P, Deckert J, Arolt V, Zwanzger P, Baune BT (2015) Pharmacoepigenetics of depression: no major influence of MAO-A DNA methylation on treatment response. Journal of neural transmission 122:99-108.

Ziegler C, Richter J, Mahr M, Gajewska A, Schiele MA, Gehrmann A, Schmidt B, Lesch KP, Lang T, Helbig-Lang S, Pauli P, Kircher T, Reif A, Rief W, Vossbeck-Elsebusch AN, Arolt V, Wittchen HU, Hamm AO, Deckert J, Domschke K (2016) MAOA gene hypomethylation in panic disorder-reversibility of an epigenetic risk pattern by psychotherapy. Transl Psychiatry 6:e773.

Ziegler C et al. (2018) Monoamine Oxidase A Gene Methylation and Its Role in Posttraumatic Stress Disorder: First Evidence from the South Eastern Europe (SEE)-PTSD Study. Int J Neuropsychopharmacol 21:423-432.

**Supplementary Tables**

**Table S1: Mean *MAOA* promoter / exon I / intron I region DNA methylation levels in females**

|  | **Season** | | | | | **Health Status** | | | | | **uVNTR** | | | | |
| --- | --- | --- | --- | --- | --- | --- | --- | --- | --- | --- | --- | --- | --- | --- | --- |
|  | **Spring / Summer** | | **Autumn / Winter** | | **p** | **PAT** | | **HC** | | **p** | **High** | | **Low** | | **p** |
|  | N | Mean ± SD | N | Mean ± SD |  | N | Mean ± SD | N | Mean ± SD |  | N | Mean ± SD | N | Mean ± SD |  |
| Avg. | 24 | 0.50 ± 0.09 | 20 | 0.44 ± 0.07 | **0.03*** | 18 | 0.47 ± 0.07 | 26 | 0.48 ± 0.10 | **0.70** | 22 | 0.47 ± 0.07 | 22 | 0.48 ± 0.10 | **0.70** |
| CpG1 | 19 | 0.59 ± 0.22 | 18 | 0.58 ± 0.25 | **0.99** | 15 | 0.71 ± 0.23 | 22 | 0.50 ± 0.20 | **<0.01*** | 18 | 0.53 ± 0.18 | 19 | 0.63 ± 0.27 | **0.22** |
| CpG2 | 24 | 0.35 ± 0.11 | 20 | 0.24 ± 0.08 | **<0.01*** | 18 | 0.27 ± 0.10 | 26 | 0.32 ± 0.12 | **0.16** | 22 | 0.31 ± 0.09 | 22 | 0.29 ± 0.13 | **0.61** |
| CpG3 | 23 | 0.50 ± 0.16 | 20 | 0.39 ± 0.12 | **0.04*** | 17 | 0.37 ± 0.13 | 26 | 0.49 ± 0.15 | **<0.01*** | 22 | 0.44 ± 0.15 | 21 | 0.45 ± 0.15 | **0.89** |
| CpG4 | 23 | 0.47 ± 0.12 | 20 | 0.42 ± 0.10 | **0.46** | 17 | 0.47 ± 0.11 | 26 | 0.44 ± 0.12 | **0.37** | 22 | 0.46 ± 0.10 | 21 | 0.44 ± 0.12 | **0.51** |
| CpG5 | 24 | 0.25 ± 0.12 | 20 | 0.18 ± 0.07 | **0.05*** | 18 | 0.21 ± 0.09 | 26 | 0.23 ± 0.11 | **0.48** | 22 | 0.22 ± 0.08 | 22 | 0.22 ± 0.12 | **0.45** |
| CpG6 | 24 | 0.47 ± 0.13 | 20 | 0.40 ± 0.12 | **0.09** | 18 | 0.44 ± 0.12 | 26 | 0.44 ± 0.14 | **0.66** | 22 | 0.44 ± 0.12 | 22 | 0.44 ± 0.14 | **0.58** |
| CpG7 | 24 | 0.57 ± 0.14 | 20 | 0.47 ± 0.12 | **0.04*** | 18 | 0.51 ± 0.12 | 26 | 0.54 ± 0.15 | **0.46** | 22 | 0.54 ± 0.12 | 22 | 0.52 ± 0.16 | **0.53** |
| CpG8 | 24 | 0.44 ± 0.15 | 20 | 0.38 ± 0.11 | **0.18** | 18 | 0.39 ± 0.13 | 26 | 0.42 ± 0.14 | **0.42** | 22 | 0.40 ± 0.09 | 22 | 0.42 ± 0.17 | **0.96** |
| CpG9 | 24 | 0.62 ± 0.11 | 20 | 0.52 ± 0.11 | **0.02*** | 18 | 0.56 ± 0.11 | 26 | 0.59 ± 0.13 | **0.73** | 22 | 0.59 ± 0.08 | 22 | 0.56 ± 0.16 | **0.31** |
| CpG10 | 24 | 0.58 ± 0.11 | 20 | 0.54 ± 0.11 | **0.38** | 18 | 0.57 ± 0.10 | 26 | 0.56 ± 0.12 | **0.28** | 22 | 0.58 ± 0.07 | 22 | 0.55 ± 0.14 | **0.33** |
| CpG11 | 24 | 0.18 ± 0.09 | 20 | 0.17 ± 0.08 | **0.93** | 18 | 0.16 ± 0.09 | 26 | 0.18 ± 0.08 | **0.28** | 22 | 0.15 ± 0.07 | 22 | 0.20 ± 0.09 | **0.14** |
| CpG12 | 24 | 0.99 ± 0.02 | 20 | 0.99 ± 0.04 | **1.00** | 18 | 0.99 ± 0.04 | 26 | 0.99 ± 0.03 | **1.00** | 22 | 0.99 ± 0.04 | 22 | 0.10 ± 0.03 | **0.29** |
| CpG13 | 24 | 0.53 ± 0.16 | 20 | 0.47 ± 0.18 | **0.18** | 18 | 0.49 ± 0.17 | 26 | 0.51 ± 0.17 | **0.67** | 22 | 0.53 ± 0.14 | 22 | 0.48 ± 0.19 | **0.29** |

**Table S1:** Mean *MAOA* promoter / exon I / intron I region DNA methylation levels (%) in females, average and CpG-specific, split by health status, variable number tandem repeats, genotype and season. P-values for independent group comparisons (two-sided Mann-Whitney U test) are indicated in a separate column (* = significant at p_uncorr_ < 0.05). **Abbreviations:** Avg., average; PAT, patients; HC, healthy controls; uVNTR, upstream variable number of tandem repeats

**Table S2: Mean MAOA promoter / exon I / intron I region DNA methylation levels in males**

|  | **Season** | | | | | **Health Status** | | | | | **uVNTR** | | | | |
| --- | --- | --- | --- | --- | --- | --- | --- | --- | --- | --- | --- | --- | --- | --- | --- |
|  | **Spring / Summer** | | **Autumn / Winter** | | **p** | **PAT** | | **HC** | | **p** | **High** | | **Low** | | **p** |
|  | N | Mean ± SD | N | Mean ± SD |  | N | Mean ± SD | N | Mean ± SD |  | N | Mean ± SD | N | Mean ± SD |  |
| Avg. | 7 | 0.16 ± 0.09 | 22 | 0.12 ± 0.03 | **0.26** | 11 | 0.15 ± 0.07 | 18 | 0.12 ± 0.03 | **0.06** | 22 | 0.13 ± 0.06 | 7 | 0.13 ± 0.02 | **0.823** |
| CpG1 | 7 | 0.04 ± 0.10 | 19 | <0.01 ± <0.01 | **0.27** | 9 | 0.03 ± 0.09 | 17 | <0.01 ± <0.01 | **0.35** | 20 | 0.01 ± 0.06 | 6 | <0.01 ± <0.01 | **1.00** |
| CpG2 | 7 | 0.02 ± 0.06 | 22 | 0.00 ± 0.01 | **0.66** | 11 | 0.02 ± 0.05 | 18 | <0.01 ± <0.01 | **0.24** | 22 | 0.01 ± 0.04 | 7 | <0.01 ± <0.01 | **0.512** |
| CpG3 | 7 | 0.22 ± 0.11 | 21 | 0.20 ± 0.18 | **0.52** | 11 | 0.25 ± 0.22 | 17 | 0.18 ± 0.12 | **0.67** | 21 | 0.18 ± 0.14 | 7 | 0.28 ± 0.22 | **0.278** |
| CpG4 | 7 | 0.05 ± 0.14 | 22 | <0.01 ± <0.01 | **0.24** | 11 | 0.04 ± 0.11 | 18 | <0.01 ± <0.01 | **0.14** | 22 | 0.02 ± 0.08 | 7 | 0.01 ± 0.02 | **1.00** |
| CpG5 | 7 | 0.03 ± 0.06 | 22 | 0.02 ± 0.06 | **0.70** | 11 | 0.05 ± 0.09 | 18 | 0.01 ± 0.03 | **0.03*** | 22 | 0.03 ± 0.07 | 7 | 0.01 ± 0.02 | **0.870** |
| CpG6 | 7 | 0.06 ± 0.16 | 22 | 0.00 ± 0.02 | **0.24** | 11 | 0.05 ± 0.12 | 18 | <0.01 ± <0.01 | **0.14** | 22 | 0.02 ± 0.09 | 7 | <0.01 ± <0.01 | **1.00** |
| CpG7 | 7 | 0.07 ± 0.15 | 22 | 0.02 ± 0.05 | **0.67** | 11 | 0.06 ± 0.13 | 18 | 0.01 ± 0.03 | **0.14** | 22 | 0.04 ± 0.09 | 7 | 0.02 ± 0.04 | **0.948** |
| CpG8 | 7 | 0.05 ± 0.10 | 22 | <0.01 ± <0.01 | **0.05** | 11 | 0.03 ± 0.08 | 18 | 0.01 ± 0.03 | **0.45** | 22 | 0.02 ± 0.06 | 7 | <0.01 ± <0.01 | **0.557** |
| CpG9 | 7 | 0.10 ± 0.17 | 22 | 0.03 ± 0.06 | **0.56** | 11 | 0.09 ± 0.14 | 18 | 0.02 ± 0.05 | **0.18** | 22 | 0.06 ± 0.11 | 7 | <0.01 ± <0.01 | **0.593** |
| CpG10 | 7 | 0.08 ± 0.16 | 22 | 0.01 ± 0.02 | **0.31** | 11 | 0.04 ± 0.13 | 18 | 0.01 ± 0.04 | **0.53** | 22 | 0.03 ± 0.09 | 7 | <0.01 ± <0.01 | **0.569** |
| CpG11 | 7 | 0.05 ± 0.06 | 22 | 0.01 ± 0.03 | **0.35** | 11 | 0.02 ± 0.04 | 18 | 0.02 ± 0.04 | **0.84** | 22 | 0.02 ± 0.04 | 7 | 0.01 ± 0.03 | **0.883** |
| CpG12 | 7 | 0.95 ± 0.09 | 22 | 0.91 ± 0.09 | **0.14** | 11 | 0.94 ± 0.07 | 18 | 0.91 ± 0.10 | **0.51** | 22 | 0.91 ± 0.10 | 7 | 0.94 ± 0.04 | **0.832** |
| CpG13 | 7 | 0.38 ± 0.07 | 22 | 0.36 ± 0.14 | **0.68** | 11 | 0.37 ± 0.12 | 18 | 0.36 ± 0.13 | **0.96** | 22 | 0.37 ± 0.13 | 7 | 0.35 ± 0.11 | **0.644** |

**Table S2:** Mean *MAOA* promoter / exon I / intron I region DNA methylation levels (%) in males, average and CpG-specific, split by health status, variable number tandem repeats, genotype and season. P-values for independent group comparisons (two-sided Mann-Whitney U test) are indicated in a separate column (* = significant at p_uncorr_ < 0.05). **Abbreviations:** Avg., average; PAT, patients; HC, healthy controls; uVNTR, upstream variable number of tandem repeats
